# Features of IgG4-related sclerosing mesenteritis: A Chinese cohort study and literature review

**DOI:** 10.1515/rir-2025-0026

**Published:** 2025-12-27

**Authors:** Jialei Zhang, Yuxue Nie, Jingna Li, Nianyi Zhang, Xinli Yang, Jialing Jiang, Yifei Wang, Qinhuan Luo, Jiaxin Zhou, Linyi Peng, Wen Zhang

**Affiliations:** Department of Rheumatology and Clinical Immunology, National Clinical Research Center for Dermatologic and Immunologic Diseases (NCRC-DID), The Ministry of Education Key Laboratory, Peking Union Medical College Hospital, Chinese Academy of Medical Sciences, Peking Union Medical College, Beijing, China; Department of Internal medicine, Peking Union Medical College Hospital, Chinese Academy of Medical Sciences, Peking Union Medical College, Beijing, China

**Keywords:** IgG4-related disease, sclerosing mesenteritis, cohort study, PET-CT, non-surgical treatment

## Abstract

**Objectives:**

This study aimed to summarize the features of IgG4-related sclerosing mesenteritis (IgG4-SM) based on both our large prospective IgG4-related disease (IgG4-RD) cohort and literature-reported IgG4-SM cases.

**Methods:**

We applied a mixed-methods approach, integrating data from 29 patients with IgG4-SM and 87 matched IgG4-RD patients without mesenteritis, all enrolled in a prospective cohort of IgG4-RD from Peking Union Medical College Hospital (PUMCH) since 2011. Additionally, we included 32 IgG4-SM cases reported in public databases since 2011. Demographic, clinical, laboratory, imaging and treatment data were systematically compared.

**Results:**

Compared with IgG4-RD without mesenteritis, IgG4-SM predominantly affected elderly males and accompanied with more organ involvement (*P* = 0.019) and elevated responder index (RI) scores and Physician Global Assessment (PGA) scores (*P* = 0.005 and *P* = 0.018, respectively). Compared to the literature-reported cases, IgG4-SM patients in our cohort exhibited a lower prevalence of abdominal pain (*P* < 0.001) but a higher frequency of submandibular gland (*P* < 0.001) and lacrimal gland enlargement (*P* < 0.001). Imaging features included a spectrum of mesenteric abnormalities, ranging from misty opacities to soft-tissue masses on computed tomography (CT), with significantly increased ^18^F-fluorodeoxyglucose (^18^F-FDG) uptake on positron emission tomography-computed tomography (PET-CT). Despite divergent treatments (glucocorticoids/immunosuppressants in our cohort *vs*. surgical resection in reported cases, *P* < 0.001), long-term relapse rates were comparable (*P* = 0.17).

**Conclusions:**

IgG4-SM is a significant but under-recognized manifestation of IgG4-RD, characterized by a predilection for elderly males, multi-organ involvement, higher disease activity and distinct imaging features. Systemic radiological evaluation, including PET-CT could assist diagnosis and treatment monitoring. Glucocorticoid-based therapy achieved outcomes comparable to surgery, challenging the necessity of invasive interventions. This study expands the clinical spectrum of IgG4-SM and advocates for personalized, non-surgical management strategies in most cases.

## Introduction

IgG4-related disease (IgG4-RD) is a chronic fibroinflammatory disorder of unknown etiology, characterized by dense lymphoplasmacytic infiltration with abundant IgG4-positive plasma cells and distinctive fibrosis.^[1]^ Patients typically present with organomegaly or fibrosis involving the lacrimal glands, submandibular glands, pancreas, biliary tract, lungs, kidneys, and lymph nodes.^[2,3]^ In contrast, involvement of the mesentery is relatively rare in the current literature.^[4]^

Due to its low prevalence, IgG4-related sclerosing mesenteritis (IgG4-SM) remains relatively understudied. In addition to the rarity of IgG4-SM, the nonspecific clinical manifestations, imaging features that mimic malignant tumors, and the lack of specific biomarkers pose challenges to its diagnosis. In the 2019 American College of Rheumatology/European League Against Rheumatism classification criteria for IgG4-RD, mesentery is not listed as a “a typical organ” in the part of entry criteria.^[5]^ Current treatment strategies are primarily based on expert consensus guidelines for IgG4-RD,^[6]^ as no specific guidelines for IgG4-SM have been established to date. Moreover, the efficacy of different treatment modalities, such as corticosteroids, immunosuppressive agents, and surgical resection, remain unclear.

Current evidence on IgG4-SM is mainly limited to case reports and small case series, showing highly variable clinical characteristics and outcomes.^[7, 8, 9, 10, 11, 12]^ In this study, we aimed to summarize the clinical manifestations of IgG4-SM by combining and comparing cases from a prospective cohort of IgG4-RD at Peking Union Medical College Hospital (PUMCH) with previously reported cases in the literature. Our objective is to describe the clinical, laboratory and radiological findings, as well as treatments and outcomes of IgG4-SM, thus to provide new insights into mesentery involvement in IgG4-RD.

## Patients and Methods

### Study Population

**Figure 1:**
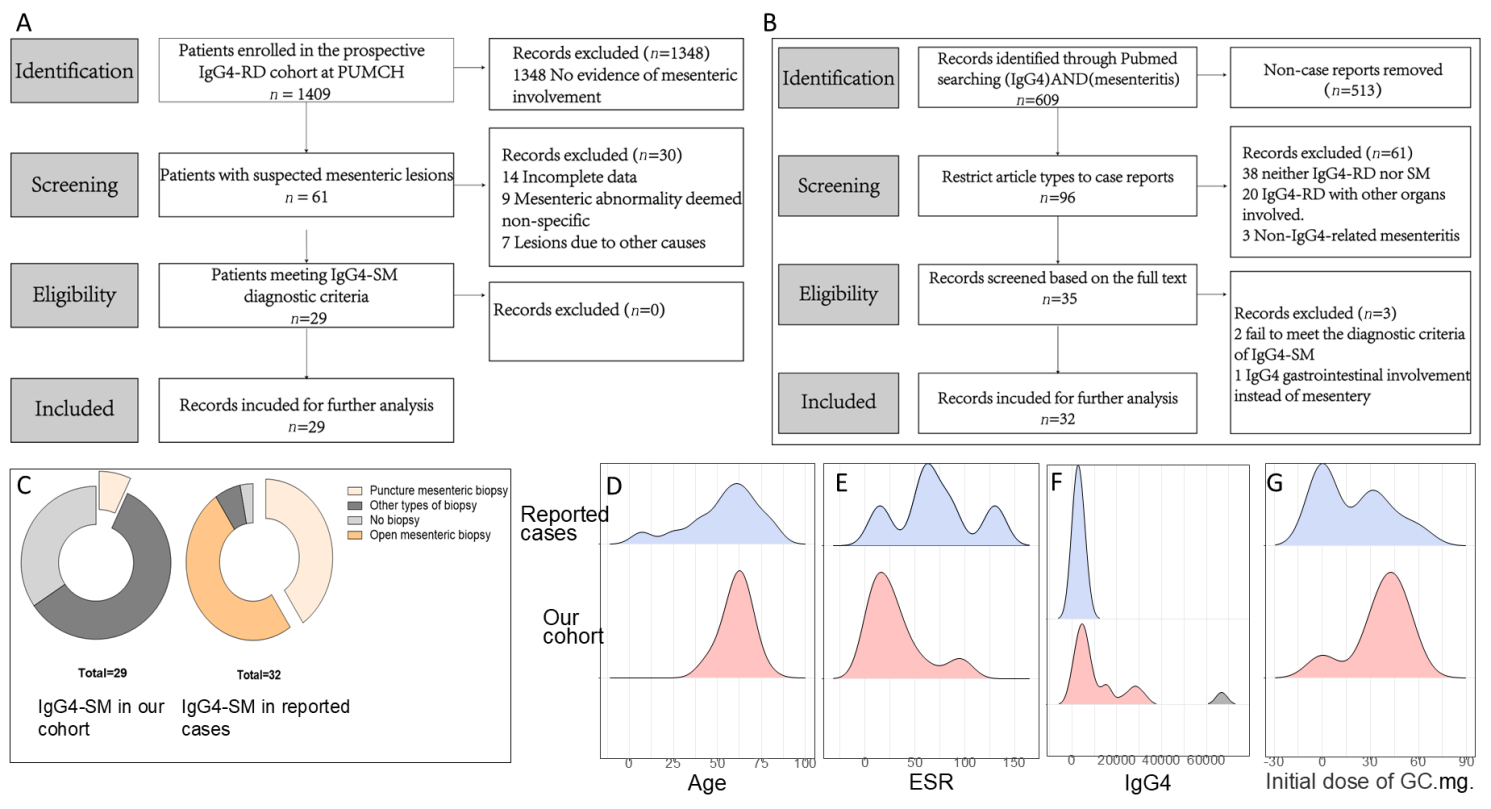
Comparison of clinical characteristics between our cohort and reported cases of IgG4-SM. (A) Flowchart of case selection for IgG4-SM from the prospective IgG4-RD cohort at PUMCH. (B) Flowchart of case selection for IgG4-SM from the literature. (C) Proportion of biopsy types in patients of the two groups. (D) Age distribution of the two groups. (E) Comparison of erythrocyte sedimentation rate (ESR, mm/h) of the two groups (P = 0.002). (F) Comparison of IgG4 levels (mg/dL) of the two groups (P < 0.001). (G) Initial dose of glucocorticoid (mg/d).

A total of 1, 409 patients were enrolled in the prospective IgG4-RD cohort established at Peking Union Medical College Hospital (PUMCH; ClinicalTrials. gov identifier NCT01670695). All patients in this cohort were consecutively enrolled at PUMCH between January 2011 and October 2024. No personally identifiable information was accessible after data collection. All patients in the cohort met the 2019 American College of Rheumatology/European League Against Rheumatism (ACR/EULAR) classification criteria for IgG4-RD and/or the 2020 revised Comprehensive Diagnostic Criteria for IgG4-RD. To identify patients with IgG4-SM within the prospective cohort, we systematically screened all 1, 409 patients with suspected mesenteric lesions based on imaging reports, which identified 61 potential cases. These records were then rigorously assessed against the predefined diagnostic criteria for IgG4-SM. A total of 32 records were excluded for: incomplete data; non-specific abnormalities; or other causes (*e.g*., malignancy, infection). After this eligibility assessment, 29 patients confirmed to have IgG4-SM were included in the final analysis, as illustrated in Figure 1A. For comparison, patients without IgG4-SM were randomly selected from the same prospective IgG4-RD cohort at a 1:3 ratio. The study was conducted in accordance with the Declaration of Helsinki and was approved by the Ethics Committee of Peking Union Medical College Hospital (approval number S-442). Written informed consent was obtained from all patients.

This study employed a mixed-methods approach since we combined cases identified in PUMCH cohort with those previously reported. Therefore, we included reported cases with IgG4-SM available in the public database. A systematic literature search was performed in the PubMed database for articles published between 2000 and 2024, using the keywords “IgG4” and “sclerosing mesenteritis, ” which initially identified 609 records. After restricting the article type to case reports, 96 records underwent title and abstract screening. Following a full-text review of the remaining articles for eligibility, 32 cases from the literature were ultimately included for analysis (see Figure 1B).

### Diagnosis of IgG4-SM

The diagnosis of IgG4-SM was based on the following criteria:(1) fulfillment of 2020 revised Comprehensive Diagnostic Criteria or the 2019 ACR/EULAR classification criteria for IgG4-RD. (2) imaging evidence of mesenteric involvement, such as hazy shadows, irregular soft tissue masses, or increased ^18^F-fluorodeoxyglucose (^18^F-FDG) uptake on positron emission tomography-computed tomography (PET-CT), and/or pathological evidence of focal or diffuse fibrosis and inflammatory changes in the small bowel mesentery.^[7,13]^ (3) exclusion of other causes, such as malignancy or infection.

### Data Collection and Follow-up

The demographic and clinical characteristics of the study cohort were collected, including age, sex, disease duration, allergy history, symptom onset, affected organs, therapeutic strategy and follow-up duration. All laboratory test results were obtained at baseline, corresponding to the patients’ first clinic visit before the initiation of treatment. Laboratory data included complete blood count (CBC), liver function, renal function, erythrocyte sedimentation rate (ESR), C-reactive protein (CRP), serum IgG, IgA, IgM, total IgE, IgG subclass levels, and complement 3 and 4. When measuring serum IgG4 levels, a standardized procedure was used to mitigate the prozone effect.^[14]^ All patients underwent abdominal imaging examinations, including computed tomography (CT), magnetic resonance imaging (MRI), or PET-CT, as needed. Pathological biopsies of affected organs were performed in some patients for the diagnosis of IgG4-RD. Patients from the PUMCH cohort were followed until relapse, lost to follow-up, or March 2025, whichever occurred first.

For cases identified in the literature, key data was extracted, including demographics, clinical manifestations, laboratory findings, treatment modalities, and prognosis.

### Disease Activity and Clinical Response

The IgG4-RD Responder Index (RI) score was used to evaluate disease activity with a score of 0–3 for each affected organ per visit.^[15]^ The Physician Global Assessment (PGA) is a subjective evaluation of overall disease activity. Disease relapse was defined as the recurrence or worsening of affected organs, new symptoms, or organ/site-specific radiological manifestations.^[16]^ Clinical and radiological remission of IgG4-SM were defined as follow: (1) Complete remission: Complete disappearance of symptoms (clinical) or lesions on imaging (radiological); (2) Partial remission: Improvement in symptoms (clinical) or reduction in lesion size on imaging (radiological); (3) No remission: No significant change in symptoms (clinical) or lesions on imaging (radiological); (4) Worsening: Increase in symptom severity (clinical) or enlargement of lesions on imaging (radiological).^[17]^

### Statistical Analysis

Variables were described using mean ± standard deviation or median with IQR (interquartile range) based on whether the data were normally distributed. Normally distributed data between two groups were analyzed using independent samples *t*-tests, while non-normally distributed data were analyzed using rank-sum tests. Categorical data were analyzed using *chi-square* tests or Fisher’s exact tests. Kaplan-Meier curves and Log-Rank tests were used to calculate and compare the cumulative relapse rates among different groups. Statistical analyses were performed using SPSS (version 23.0, IBM), R Studio (version 4.3.1, https://www.r-project.org), Adobe Illustrator CC 2015 (Adobe, Cal, USA), and Prism software version 6.1 (GraphPad Software, La Jolla, CA, USA). A two-tailed *P*-value < 0.05 was considered statistically significant.

## Results

### Demographic and Clinical Characteristics

This study included 29 patients from PUMCH and 32 from the literature, totally 61 patients with IgG4-SM. The 29 IgG4-SM patients in PUMCH cohort were assigned as the case group, an additional 87 IgG4-RD patients without sclerosing mesenteritis were randomly selected at a 1: 3 ratio from the IgG4-RD cohort in PUMCH as controls, with no matching geographical features and other clinical parameters, to avoid bias. The demographic and clinical characteristics of IgG4-SM patients are summarized in Table 1.

**Table 1:** Baseline features of IgG4-RD patients with/without sclerosing mesenteritis

Parameters	IgG4 related SM (*n* = 29)	IgG4-RD without SM (*n* = 87)	*P*-value
Male, *n* (%)	17 (58.6%)	50 (57.5%)	1
Age of diagnosis, years (IQR)	63.0 [56.0;65.0]	56.0 [47.0;61.5]	0.001^*^
Proportion of Males ≥60 Years	12 (41.4%)	17 (19.5%)	0.019^*^
Disease duration, month (IQR)	15.0 [6.00;24.0]	12.0 [4.00;36.0]	0.362
Involved organs, *n* (IQR)	4.00 [3.00;6.00]	3.00 [2.00;5.00]	0.019^*^
Allergy history, *n* (%)	16 (55.2%)	44 (53.0%)	1
Other involved organs, *n* (%)			
Pancreas	14 (48.3%)	33 (37.9%)	0.445
Submandibular glands	16 (55.2%)	37 (42.5%)	0.333
Lacrimal glands	16 (55.2%)	36 (41.4%)	0.281
Cholangitis	5 (17.2%)	19 (21.8%)	0.791
Parotid glands	6 (20.7%)	10 (11.5%)	0.225
Retroperitoneum	6 (20.7%)	12 (13.8%)	0.384
Lung	13 (44.8%)	21 (24.1%)	0.06
Kidney	5 (17.2%)	11 (12.6%)	0.542
Lymph nodes	11 (37.9%)	35 (40.2%)	1
Paranasal sinus	10 (34.5%)	20 (23.0%)	0.327
Baseline lgG4-RD RI	8.00 [6.00;10.0]	6.00 [4.00;10.0]	0.005^*^
Baseline PGA	6.26 (1.63)	5.22 (2.45)	0.018^*^

SM: sclerosing mesenteritis; IgG4-RD RI: IgG4-RD responder index; PGA: physician’ s global assessment; *statistical significance.

In the overall cohort of IgG4-RD from PUMCH, the prevalence of IgG4-SM was 2.06% (29/1409), highlighting the rarity of sclerosing mesenteritis in IgG4-RD. The median age at diagnosis of IgG4-SM patients was 63 years (IQR 56–65), significantly higher than that of IgG4-RD patients without sclerosing mesenteritis (median 56, IQR 47–61, *P* = 0.001). Additionally, the IgG4-SM group had a higher proportion of males aged ≥ 60 years (41.4% *vs*. 19.5%, *P* = 0.019). IgG4-SM patients also presented with a greater number of involved organs (median 4, IQR 3–6) compared to those IgG4-RD patients without IgG4-SM (median 3, IQR 2–5, *P* = 0.019). However, there were no significant differences in the involvement of specific organs, such as the pancreas, lacrimal glands, and submandibular glands, between the two groups. Furthermore, IgG4-SM patients showed significantly higher IgG4-RD RI scores (8.00 [6.00–10.0] *vs*. 6.00 [4.00–10.0], *P* = 0.005) and PGA scores (6.26 ± 1.63 *vs*. 5.22 ± 2.45, *P* = 0.018) compared to those IgG4-RD patients without IgG4-SM.

We further compared the patients with IgG4-SM in our cohort to those identified through our systematic literature review (*n* = 32). Compared to the reported cases, no significant differences were observed in sex or age distribution between the two groups (Table 1, Figure 1D). In the aspect of the symptoms, less patients in our cohort presented with abdominal pain compared to reported cases (20.7% *vs*. 68.8%, *P* < 0.001). In contrast to the cases in the literature, who did not report symptoms of swollen salivary glands, patients in our cohort of exhibited considerable proportions to have enlargement of submandibular glands (44.8% *vs*. 0%, *P* < 0.001) and lacrimal glands (51.7% *vs*. 0%, *P* < 0.001). Furthermore, regarding biopsy types, the overall biopsy rate was lower in our cohort compared to that in the literature (65% *vs*. 97%, *P* < 0.001), with the majority undergoing biopsies of other affected organs and only a minority undergoing mesenteric biopsies (2/29 in our cohort), all of which were fine-needle aspirations. In contrast, in the reported cases, most of them (29/32) received mesenteric biopsies, where 21 received open surgery and 8 received core-needle aspiration. This contrast indicated the under-recognition of mesenteric involvement in IgG4-RD since its rarity. The detailed types of biopsies are shown in Figure 1C.

### Baseline Laboratory Features

Baseline laboratory features (before treatment) of the 29 IgG4-SM patients and 87 IgG4-RD patients without sclerosing mesenteritis are summarized in Table 2. No significant differences were observed between the two groups in terms of hemoglobin (Hb), white blood cell count (WBC), high-sensitivity C-reactive protein (hsCRP), total IgG, IgA, IgM, IgG subclasses (IgG1, IgG2, IgG3), total IgE, complement C3, and C4 levels.

**Table 2:** Laboratory parameters of IgG4-RD patients with/without sclerosing mesenteritis

Parameters	IgG4 related SM (*n* = 29)	IgG4-RD without SM (*n* = 87)	*P*-value
Hgb (g/L)	134 (12.9)	136 (16.2)	0.723
WBC (109/L)	6.54 [5.13;7.96]	6.96 [5.54;8.59]	0.311
Eos (%), M (IQR)	0.28 [0.10;0.43]	0.12 [0.05;0.36]	0.122
ESR (mm/h), M (IQR)	22.5 [11.2;39.5]	16.0 [8.00;34.0]	0.167
hsCRP (mg/L), M (IQR)	2.56 [0.97;5.97]	1.67 [0.60;3.87]	0.195
IgG (g/L)	17.1 [15.0;28.8]	17.8 [13.4;21.8]	0.186
IgA (g/L)	1.92 [1.54;2.73]	1.77 [1.30;2.50]	0.41
IgM (g/L), M (IQR)	0.73 [0.52;0.99]	0.81 [0.61;1.19]	0.271
IgG1 (mg/L)	9980 [8084;12520]	8860 [7415;10300]	0.172
IgG2 (mg/L)	4465 [2834;6278]	4910 [3990;6582]	0.183
IgG3 (mg/L), M (IQR)	505 [342;1017]	345 [224;666]	0.054
IgG4 (mg/L), M (IQR)	6580 [3950;15800]	6390 [2405;13200]	0.388
T-IgE (KU/L), M (IQR)	507 [134;950]	314 [94.0;799]	0.363
C3 (g/L)	0.97 (0.27)	1.05 (0.28)	0.174
C4 (g/L)	0.15 [0.10;0.22]	0.17 [0.13;0.25]	0.166

Hgb: hemoglobin; WBC: white blood cell count; Eos: eosinophils; ESR: erythrocyte sedimentation rate; hsCRP: high-sensitivity C-reactive protein; IgG: Immunoglobulin G; IgA: Immunoglobulin A; IgM: Immunoglobulin M; IgG1: Immunoglobulin G1; IgG2: Immunoglobulin G2; IgG3: Immunoglobulin G3; IgG4: Immunoglobulin G4; T-IgE: total Immunoglobulin E; C3: Complement component 3; C4: Complement component 4.

Compared with the 32 IgG4-SM in reported cases (see Table 3 or Figure 1E-F), patients in our cohort exhibited significantly lower levels of ESR (22.5 mm/h [11.2; 39.5] *vs*. 74.5 mm/h [53.5; 101], *P* = 0.002) and hsCRP (2.56 mg/L [0.97; 5.97] *vs*. 28.0 mg/L [22.0; 67.0], *P* < 0.001). Additionally, IgG4 levels were significantly higher in our cohort compared to the reported cases (6580 mg/L [3950; 15800] *vs*. 1693 mg/L [1190; 2898], *P* < 0.001).

**Table 3:** Comparison of features of IgG4-SM in our cohort and reported cases

Parameters	IgG4 related SM (*n* = 29)	Reported cases (*n* = 32)	*P*-value
Male, n(%)	17 (58.6%)	21 (65.6%)	0.765
Age of diagnosis	63.0 [56.0;65.0]	58.0 [50.8;67.2]	0.452
Proportion of males ≥60 Years	12 (41.4%)	10 (31.3%)	0.411
Abdominal pain	6 (20.7%)	22 (68.8%)	<0.001^*^
Nausea/Vomiting	2 (6.90%)	8 (25.0%)	0.084
Fever	0 (0.00%)	3 (9.38%)	0.239
Lymph node enlargement	3 (10.3%)	1 (3.12%)	0.338
Submandibular gland enlargement	13 (44.8%)	0 (0.00%)	<0.001^*^
Lacrimal gland enlargement	15 (51.7%)	0 (0.00%)	<0.001^*^
Parotid gland enlargement	3 (10.3%)	0 (0.00%)	0.102
Nasal congestion	4 (13.8%)	0 (0.00%)	0.046^*^
ESR (mm/h)	22.5 [11.2;39.5]	74.5 [53.5;101]	0.002^*^
CRP (mg/L)	2.56 [0.97;5.97]	28.0 [22.0;67.0]	<0.001^*^
IgG4 (mg/L)	6580 [3950;15800]	1693 [1190;2898]	<0.001^*^
Surgical resection	2 (6.9%)	19 (59.4%)	<0.001^*^

ESR: erythrocyte sedimentation rate; CRP: C-reactive protein. *statistical significance.

### Imaging Features

**Figure 2:**
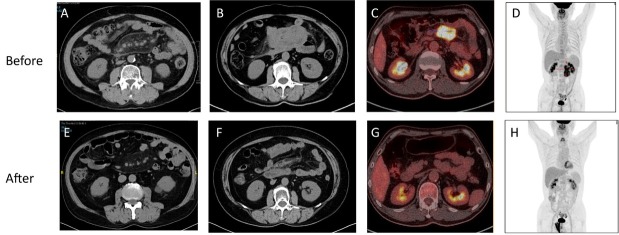
Characteristic imaging findings of IgG4-SM before and after treatment. (A) Abdominal CT axial image of a patient with mild IgG4-SM showing hazy shadows in the mesenteric region. (B) Abdominal CT axial image of a patient with severe IgG4-SM showing irregular soft tissue masses in the mesenteric region. (C-D) PET-CT images of an IgG4-SM patient showing soft tissue masses in the mesenteric region with significantly increased ^18^F-FDG uptake, indicating high metabolic activity. (E-H) Post-treatment images corresponding to (A-D) demonstrate reduced lesion size, resolved hazy shadows, decreased soft tissue masses, and diminished ^18^F-FDG uptake, indicating a favorable treatment response.

IgG4-SM exhibits characteristic imaging features. Abdominal CT axial images often reveal hazy shadows in the mesenteric region, indicative of mild inflammatory infiltration (see Figure 2A). In more severe cases, CT images may display irregular soft tissue masses, suggesting fibrosis and active inflammation (Figure 2B). PET-CT scans further reveal significant increases in ^18^F-FDG uptake within these soft tissue masses, reflecting high metabolic activity (Figure 2C-D).

Despite the potential difficulty in distinguishing IgG4-SM from malignancies on imaging, patients with IgG4-SM typically exhibit favorable radiological responses to corticosteroid therapy. Post-treatment imaging shows reduced lesion size, alleviation of hazy shadows, shrinkage of soft tissue masses, and decreased ^18^F-FDG uptake, indicating effective treatment (Figure 2E-H). Compared to previously reported cases of IgG4-SM, our cohort exhibits similar imaging characteristics.^[18]^

### Pathological Features

**Figure 3:**
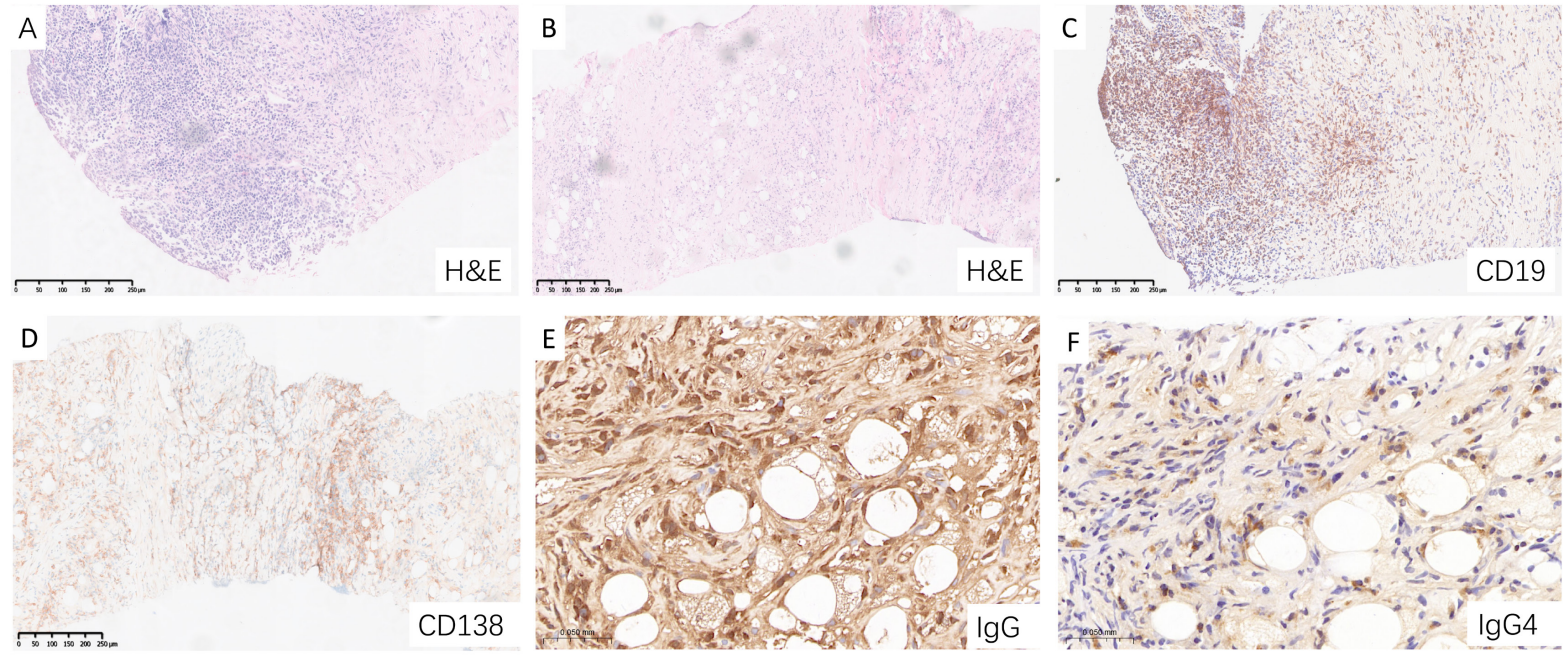
Characteristic pathological features of IgG4-SM in a representative patient. (A) Hematoxylin and eosin (H& E) staining showing dense lymphoplasmacytic infiltration. (B) H&E staining revealing prominent fibrosis and periadipose inflammation. (C) CD19 staining highlighting B cell infiltration. (D) CD138 staining demonstrating abundant plasma cell infiltration. (E) IgG staining showing dense infiltration of IgG-positive plasma cell. (F) IgG4 staining revealing scattered IgG4-positive plasma cell infiltration.

Histopathological features of IgG4-SM are consistent with those commonly affected organs. Typical mesenteric lesion from patients with IgG4-SM were shown in Figure 3, which revealed significant infiltration of lymphocytes and plasma cells, clearly demonstrated by hematoxylin and eosin (H& E) staining (Figure 3A). Fibrosis, characterized by dense collagen deposition and increased extracellular matrix, is a hallmark pathological feature of IgG4-SM, particularly in areas of active inflammation (Figure 3B).

Immunohistochemical staining showed dense infiltration of CD20^-^positive B cells, CD138^-^positive plasma cells, IgG-positive plasma cells, and IgG4-positive plasma cells (Figure 3C-F). Analysis of the staining results indicated that the ratio of IgG4-positive plasma cells to IgG-positive plasma cells (IgG4+/IgG+) exceeded 40%, with a density of IgG4-positive plasma cells greater than 10 cells per high-power field (HPF). The histopathological features observed in the mesenteric tissues from our cohort were consistent with those described in the literature-reported cases, demonstrating the characteristic lymphoplasmacytic infiltration, fibrosis, and immunohistochemical profile typical of IgG4-RD.

### Treatment and Prognosis

**Figure 4:**
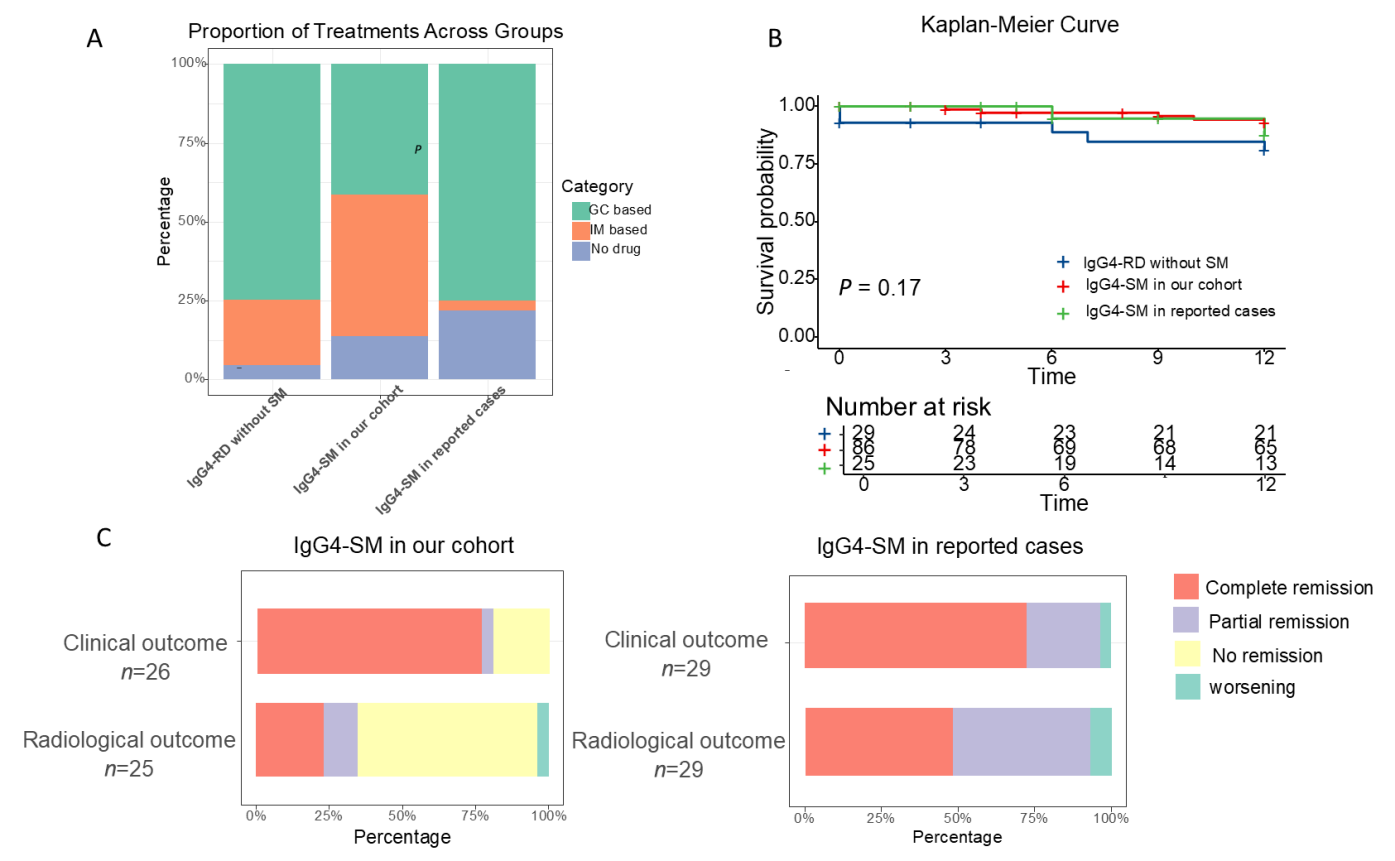
Comparison of treatment and prognosis among IgG4-RD without SM, IgG4-SM in our cohort, and IgG4-SM in reported cases. (A) Treatment modalities compared among the three groups. (B) Kaplan-Meier non-relapse survival curves for the three groups (log-rank test, P = 0.17). (C) Treatment outcomes in IgG4-SM patients from our cohort and reported cases.

In our cohort, the primary baseline treatment modality for IgG4-SM was corticosteroids, either alone or in combination with immunosuppressive agents (Figure 4A; treatment details are provided in Supplementary Table S1). Initial treatment typically involved prednisone at a dose of 30–40 mg/ day (Figure 1G). In contrast, surgical resection was less frequent in our patients compared to the reported cases (resection rate: 6.9% *vs*. 59.4%, *P* < 0.001, Table 3).

After the treatment, the majority of IgG4-SM patients in our cohort exhibited favorable clinical and radiological responses, with significant symptom alleviation and reduced lesion size on imaging (Figure 4C). However, compared to the reported cases, our cohort had a lower radiological complete response rate (23.1% *vs*. 48.3%, *P* = 0.04), which may be attributed to differences in treatment approaches.

The median follow-up time in our cohort was 20 months (IQR 6–61), significantly longer than that in reported cases (median 6 months, IQR 4–12, *P* = 0.02). Despite this difference, Kaplan-Meier analysis showed no significant differences in cumulative relapse rates among the three groups (*P* = 0.17, Figure 4B).

Seven patients were followed for ≥ 5 years, including one patient for more than 10 years. All seven patients were alive at the last follow-up (overall survival rate, 100%). Five patients (71.4%) experienced relapse during follow-up, either in previously affected organs (for example, pancreas, sinus, or mesentery) or in newly involved sites such as the retroperitoneum and mediastinal lymph nodes. The remaining two patients maintained sustained remission throughout follow-up. Detailed clinical courses of these seven patients are presented in Supplementary Table S2. These findings indicate that IgG4-SM generally demonstrates a favorable long-term outcome but may relapse several years after initial remission, particularly following glucocorticoid tapering or discontinuation.

## Discussion

This mixed-methods study characterizes the clinical features of IgG4-SM drawing on the largest prospective IgG4-RD cohort in China^[19]^ and a systematic review of published cases. Our finding reveals that IgG4-SM predominantly affects elderly males with extensive multi-organ involvement. It also presents with distinct radiological features and achieves comparable outcomes with either glucocorticoid-based therapy or surgical resection. Given the extreme rarity of IgG4-SM, integrating data from all previously reported cases allowed us to place our cohort findings into a broader clinical context and to delineate the full spectrum of this manifestation within IgG4-RD. These results provide new evidence that IgG4-SM may not always represent an isolated, surgery-indicated condition.

Our study found that IgG4-SM predominantly affects elderly males^[20]^ and is associated with greater organ involvement and higher disease activity, as indicated by RI and PGA scores. These findings suggest that IgG4-SM may represent a more systemic or aggressive phenotype within the IgG4-RD spectrum, rather than a localized manifestation. Although organ involvement patterns were similar, the total disease burden was higher in IgG4-SM patients, underscoring the need to assess for mesenteric involvement even in patients with typical IgG4-RD features.^[4]^

Compared to literature-reported cases, patients in our cohort had fewer abdominal symptoms but a higher frequency of submandibular and lacrimal gland enlargement. In published reports, abdominal pain often emerged as the initial symptom, likely reflecting a selection bias toward symptom-driven diagnoses.^[10]^ In contrast, many IgG4-SM cases in our prospective cohort were incidentally identified during systemic evaluation for IgG4-RD, suggesting that mesenteric involvement may often be silent and under-recognized. These findings underscore the value of comprehensive imaging in revealing the full clinical spectrum of IgG4-SM, ranging from asymptomatic to overtly symptomatic disease. Clinicians should be aware that IgG4-SM may not always present with typical abdominal complaints and may require proactive imaging assessment, especially in patients with high systemic disease burden.

Radiologically, IgG4-SM in our cohort exhibited a spectrum of CT findings, ranging from subtle misty opacities with poorly defined margins to irregular soft-tissue masses in the mesenteric region, likely reflecting different stages or intensities of fibroinflammatory infiltration. Notably, in contrast to many reported cases that highlight bulky mesenteric tumors,^[4,9, 10, 11]^ our data reveal that IgG4-SM can also present with subtle or diffuse radiological abnormalities that may be easily overlooked. This broader imaging spectrum underscores the potential for underdiagnosis in routine clinical practice.

^18^F-FDG PET/CT further demonstrated increased metabolic activity within mesenteric lesions, with elevated standardized uptake value (SUV) consistent with active inflammation—findings in line with previous reports.^[18]^ Following glucocorticoid-based therapy, both lesion size and FDG uptake significantly declined, supporting the utility of PET/CT not only in initial detection but also in monitoring therapeutic response.^[21]^ By capturing metabolic changes beyond structural alterations, PET/CT complements conventional imaging and may facilitate the identification of subclinical or atypical mesenteric involvement. Its incorporation into routine evaluation could improve diagnostic accuracy, inform treatment decisions, and reduce unnecessary surgical interventions.

In our cohort, glucocorticoid-based therapy-either alone or in combination with immunosuppressants-was the primary treatment for IgG4-SM, reflecting a clinical preference for less invasive and more tolerable pharmacologic approaches.^[22]^ In contrast, surgical resection was infrequently utilized, differing markedly from the reported cases, where surgery was often employed for both diagnostic clarification and therapeutic debulking.^[4]^ This contrast highlights the diagnostic challenge of IgG4-SM, as the non-specific radiological appearance and difficulty in obtaining adequate biopsy samples may prompt more aggressive interventions in real-world practice.^[1]^ In our cohort, however, biopsies, when performed, were typically conducted *via* fine-needle aspiration rather than open surgical procedures, thereby minimizing patient burden.

Although the complete remission rate in our cohort was lower than in reported cases-possibly due to the mechanical removal of lesions during surgery-the overall remission and cumulative relapse rates were comparable. These findings suggest that differences in treatment strategies, particularly the use of glucocorticoid-based versus surgery-based approaches, did not substantially affect long-term outcomes. For many patients, glucocorticoid-based medical therapy may be sufficient to achieve disease control, reinforcing the feasibility of a non-surgical approach in managing IgG4-SM. Overall, our results indicate that treatment modality per se is unlikely to be the major determinant of prognosis in IgG4-SM. Our results challenge the traditional notion that IgG4-SM necessitates surgical intervention and emphasize the importance of early recognition, radiologic monitoring, and individualized treatment planning to avoid overtreatment.

Despite its strengths, this study has several limitations. First, although the sample size of IgG4-SM patients is larger than in previous reports, the rarity of the condition means that the sample size still remains relatively small, potentially limiting the generalizability of our findings and the statistical power of some analyses. Second, the retrospective nature of the literature review introduces potential bias, as case reports often focus on more severe or atypical presentations of the disease. Third, since the diagnosis of IgG4-RD had already been confirmed through pathological examination of other involved organs, the small number of mesenteric biopsy in our cohort may limit a comprehensive characterization of the pathological features of IgG4-SM. Additionally, despite efforts to standardize the data, differences in laboratory methodologies and diagnostic criteria across institutions may have affected the comparability of our results with reported cases.

## Conclusion

In conclusion, this study describes the clinical characteristics, imaging features, and treatment outcomes of IgG4-SM based on a prospective IgG4-RD cohort in China, supplemented by a comprehensive literature review. Our findings show that IgG4-SM presents with heterogeneous manifestations and can often be managed effectively with glucocorticoid-based therapy. The observed differences in treatment strategies underscore the variability in real-world practice and the need for careful clinical assessment. Glucocorticoid-based therapy was effective for many patients in our cohort. Further research is needed to clarify the underlying pathophysiology and optimize diagnostic and therapeutic strategies for this rare but impactful condition.

## Supplementary Material

Supplementary Material Details
