# Supplementary material for: Features of IgG4-related sclerosing mesenteritis: A Chinese cohort study and literature review
Source: Rheumatol Immunol Res. 2025 Dec 27;6(4):210–9. doi: 10.1515/rir-2025-0026 (PMC12743267; doi:10.1515/rir-2025-0026)
Supplement: Supplementary file 1 — Supplementary Material Details [file rir-2025-0026_sm.pdf]

Supplementary Table S1. Treatment regimens of three patient groups: IgG4-RD without SM, IgG4-SM in our cohort, and IgG4-SM in reported cases

Baseline treatment type	Specific regimen	IgG4-RD without SM (*n* = 87)	IgG4-SM in our cohort (*n* = 29)	IgG4-SM in reported cases (*n* = 32)
GC-based therapy	GC monotherapy	18	11	8
	GC+CTX	17	2	1
	GC+MMF	11	6	1
	GC+LEF	3	1	0
	GC+RTX	3	0	0
	GC+MTX	3	0	0
	GC+AZA	2	1	0
	GC+IGU	2	0	0
	GC+TII	1	2	0
	GC+HCQ	1	0	0
	GC+HCQ+TII	1	0	0
	GC+HCQ+MMF	1	0	0
	GC+BAR	1	1	0
	GC+TAC	1	0	0
	GC+IGU+MTX	0	0	0
	GC+IGU+TII	0	0	0
	GC+HCQ+TOF	0	0	0
	GC+THD	0	0	1
IMs-based therapy	IGU alone	7	1	0
	MTX alone	2	0	0
	RTX alone	2	0	0
	AZA alone	1	0	0
	MMF alone	1	1	0
	LEF alone	1	0	0
	HCQ+CTX	1	0	0
	HCQ+TII	1	0	0
	IGU+TII	1	0	0
	IGU+MTX	1	0	0
	TII alone	0	0	0
	CTX alone	0	0	1
	HCQ alone	0	1	0
	MTX alone	0	0	0
	HCQ+TOF	0	1	0
Surgery	Partial or complete resection	0	0	20
Watchful	No active	4	1	0

waiting	treatment

Supplementary Table S2. Long-term follow-up and prognosis of seven patients with IgG4-SM (≥5 years)

No.	Organs involved at baseline	Treatment regimen	Follow-up duration (months)	Relapse (Y/N)	Time to relapse (months)	Relapsed organs / sites
1	Mesentery, submandibular gland, pancreas	Pred + TwHF	152	No	—	—
2	Submandibular, lacrimal, parotid glands, lymph nodes, sinuses, mesentery	Pred + AZA	116	Yes (×2)	54, 63	Submandibular/parotid; mesentery
3	Retroperitoneum, aorta, kidneys	Pred alone	98	Yes (×1)	76	Mesentery (new site)
4	Pancreas, sinus, mesentery	DXM + IGU	84	Yes (×1)	48	Pancreas, sinus
5	Pancreas, biliary tract, mesentery, kidneys	Pred + MMF	78	No	—	—
6	Mesentery	Pred + LEF	65	Yes (×2)	1, 66	Mesentery; mediastinal lymph nodes
7	Lacrimal, parotid, pancreas, mesentery	DXM + MMF	74	Yes (×2)	27, 54	Mesentery; retroperitoneum

Abbreviations: Pred, prednisone; AZA, azathioprine; MMF, mycophenolate mofetil; LEF, leflunomide; TwHF, Tripterygium wilfordii Hook F.; DXM, dexamethasone; IGU, iguratimod; mo, months.
